# Complement System and the Kidney: Its Role in Renal Diseases, Kidney Transplantation and Renal Cell Carcinoma

**DOI:** 10.3390/ijms242216515

**Published:** 2023-11-20

**Authors:** Francesco Lasorsa, Monica Rutigliano, Martina Milella, Matteo Ferro, Savio Domenico Pandolfo, Felice Crocetto, Simona Simone, Loreto Gesualdo, Michele Battaglia, Pasquale Ditonno, Giuseppe Lucarelli

**Affiliations:** 1 Department of Precision and Regenerative Medicine and Ionian Area-Urology, Andrology and Kidney Transplantation Unit, University of Bari “Aldo Moro”, 70124 Bari, Italy; 2 Division of Urology, European Institute of Oncology, IRCCS, 71013 Milan, Italy; 3 Department of Neurosciences and Reproductive Sciences and Odontostomatology, University of Naples “Federico II”, 80131 Naples, Italy; 4 Department of Precision and Regenerative Medicine and Ionian Area-Nephrology, Dialysis and Transplantation Unit, University of Bari “Aldo Moro”, 70124 Bari, Italy

**Keywords:** complement system, complosome, kidney, nephropathy, kidney transplantation, ischemia-reperfusion injury, renal cell carcinoma, therapy

## Abstract

The crosstalk among the complement system, immune cells, and mediators of inflammation provides an efficient mechanism to protect the organism against infections and support the repair of damaged tissues. Alterations in this complex machinery play a role in the pathogenesis of different diseases. Core complement proteins C3 and C5, their activation fragments, their receptors, and their regulators have been shown to be active intracellularly as the complosome. The kidney is particularly vulnerable to complement-induced damage, and emerging findings have revealed the role of complement system dysregulation in a wide range of kidney disorders, including glomerulopathies and ischemia-reperfusion injury during kidney transplantation. Different studies have shown that activation of the complement system is an important component of tumorigenesis and its elements have been proved to be present in the TME of various human malignancies. The role of the complement system in renal cell carcinoma (RCC) has been recently explored. Clear cell and papillary RCC upregulate most of the complement genes relative to normal kidney tissue. The aim of this narrative review is to provide novel insights into the role of complement in kidney disorders.

## 1. Introduction

The complement cascade is a component of the innate immune system and has a fundamental role in defense mechanisms against pathogens such as viruses and bacteria. It is a complex network of different proteins that circulate in the blood and are also found on cell surfaces [1,2]. The crosstalk among complement system proteins, immune cells, and mediators of inflammation provides an efficient mechanism to protect the organism against infections and support the repair of damaged tissues [3,4,5]. However, alterations in this complex machinery play a role in the pathogenesis of different diseases. The role of the complement system in a variety of inflammatory diseases is multifaceted; for instance, complement activation can greatly contribute to inflammation-mediated tissue damage, whereas inherited or acquired complement deficits strongly favor the emergence of autoimmune diseases [6,7]. The kidney is particularly vulnerable to complement-induced damage. Emerging findings have revealed the role of complement system dysregulation in a wide range of kidney disorders, including glomerulopathies, ischemia-reperfusion injury during kidney transplantation, and kidney cancers [8,9,10].

Tumor-promoting inflammation plays an important role in development of many cancers [11]. Different studies have shown that activation of the complement system is an important component of this protumorigenic process [12]. In addition, the complement system contributes to cancer progression, increasing the activity of mitogenic signaling pathways, exerting prosurvival and antiapoptotic effects, inducing angiogenesis, and facilitating cancer cell migration and invasion [13]. Renal cell carcinoma (RCC) is the most common form of malignant renal tumor, and its incidence has risen by roughly 2% annually during the past 20 years [14,15,16]. In the last years, many studies have showed that several metabolic disorders, such as obesity, metabolic syndrome, diabetes, and chronic kidney diseases, represent common risk factors for this cancer [17,18,19,20,21,22,23,24,25,26,27,28,29,30,31,32]. Moreover, RCC is one of the most immune-infiltrated tumors, and the reactive immunoflogosis can accelerate cancer progression, by enriching the tumor microenvironment (TME) with cytokines and growth factors [33]. In this scenario, complement system activation in TME can promote immunosuppression and cancer growth and can sustain angiogenesis, a hallmark of renal cancer [34,35].

In this narrative review, we provide novel insights into the role of complement in kidney disorders and the tumor-promoting effect of its activation in renal cell carcinoma.

## 2. Complement System (Components, Activation Cascades, Regulators, Local Activation)

**Figure 1:**
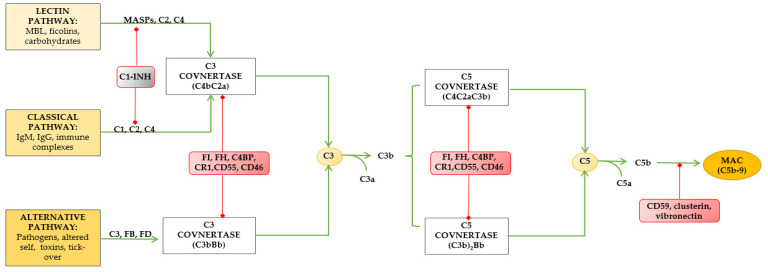
Complement system pathways. MBL: mannose-binding lectin; FI: factor I; FH: factor H; CR1: complement receptor 1; MAC: membrane-attack complex.

In 1901, Jules Bordet first described the complement system, which is regarded as one of the most ancient component of our immune system [36]. It consists of approximately 50 elements, including blood- and lymph-circulating proteins as well as membrane-bound elements (receptors and regulators). Most of the complement core elements (proteins C1 to C9, activators, and regulators) are synthetized in the liver, while receptors and membrane-bound regulatory proteins are expressed on several immune and non-immune cells [37,38]. Complement elements also function as pattern recognition receptors (PRRs), which can recognize a variety of pathogen-associated molecular patterns (PAMPs) and damage-associated molecular patterns (DAMPs). The system consists of serine proteases whose cascade activity is strictly regulated. Three different pathways have been described so far: the classical, the lectin, and the alternative pathway, the latter being phylogenetically older but later discovered (Figure 1). Although they are triggered differently, each process leads to the production of enzyme complexes that can cleave C3 complement protein [39].

The alternative pathway is constitutively activated since C3 is being continuously converted into C3b (C3 thickover) as a sentinel against pathogens. C3 is characterized by a thioester moiety that is normally hidden. When cleaved to C3b, this domain is exposed and can attach to amino or hydroxyl groups of proteins on microbes’ surface. In the absence of pathogens, the thioester group is hydrolyzed in plasma, so circulating C3b is rapidly inactivated. On a microbial or host cell surface, C3b can associate with plasma factor B. Serine protease factor D further cleaves factor B into Ba (smaller fragment) and Bb (which remains liked to C3b). C3bBb represents the alternative pathway C3 convertase, which can amplify the complement cascade since C3b can interact with Bb fragments regardless of alternative, classical, or lectin pathway initiation. Properdin (the only positive regulator of complement) stabilizes C3bBb complex on microbes, whereas it is quickly degraded by different regulators when it is assembled on normal mammalian cells. The interaction with C3 convertase itself with another C3b molecule results in the alternative pathway C5 convertase for the late events of the cascade.

The classical pathway depends on the binding of C1 complex to the Fc region of IgG or IgM that have bound antigen. C1 complex is made of C1q, C1r, and C1s subunits. Free circulating antibodies cannot initiate the pathway since each C1q subunit must bind at least two antibody heavy chains. IgG molecules only have one Fc region; hence, for C1q to interact, many IgG must be brought close together when binding multivalent antigen. Free IgM is pentameric, but it is unable to bind C1q because the Fc regions are arranged in a configuration that prevents C1q from accessing them. Antigen-binding-induced conformation changes expose C1q binding sites in Fc regions. Serine proteases C1r and C1s create a tetramer with two molecules of each protein in C1 complex. Upon binding with Ig heavy chains, C1r is activated, which in turn cleaves and activates C1s. C1q can be bound and activated by plasma proteins of the pentraxin family such as C-reactive protein (CRP), serum amyloid P (SAP), and the long pentraxin-3 (PTX3). Pentraxins recognize specific structures on microbial and apoptotic cells’ surfaces (phosphatidylethanolamine or phosphorylcholine). Their levels raise during inflammation as with other acute phase proteins synthetized in the liver. PTX3 is produced by different cell types including dendritic cells, macrophages, and endothelial cells. Then, activated C1s cleaves C4 into C4a and C4b. Similarly to C3b, because of a specific domain, C4b attaches to a cell surface or immune complex. Complexed to C4b, C2 is then activated into C2a. The resulting C4b2a complex represents the classical pathway C3 convertase: the C2a subunit catalyzes C3 proteolysis into C3a and C3b. As mentioned above, C3b deposits on cell membranes and promotes more C3 convertase via factor B. Some of the C3b fragments join to the C4b2a complex to form the C4b2a3b complex that functions as the classical pathway C5 convertase to allow the late steps of the complement cascade. The binding of microbial surface carbohydrates pattern (N-acetyl glucosamine or mannose) to soluble lectins initiates the lectin pathway in the absence of antibodies. Circulating lectins are collagen-like proteins that structurally resemble C1q. They include mannose-binding lectin (MBL) and ficolins, which then associate with MBL-associated serine proteases (MASPs: MASP1, MASP2, and MASP3). The MASP proteins, which are structurally homologous to C1r and C1s proteases, cleave C4 and C2 to trigger subsequent events that are the same as those in the classical pathway. The induction of the final steps of the complement cascade depends on C5 convertase, which cleaves C5 into a small C5a molecule and C5b, which remains on the cell surface. Transient conformation allows C5b to bind C6 and C7 proteins (C5b,6,7 complex). This complex undergoes a conformation change that exposes a hydrophobic site on C7 and draws the complex into the lipid bilayer of the cell membrane. Subsequent binding of the C5b–C6–C7 complex to C8 exposes another hydrophobic site that further anchors the 5b–8 complex into the cell membrane.

However, the C5b-8 complex (C5b, C6, C7, C8) has a limited lytic activity. The binding of C9, which polymerizes at the site of the bound C5b-8, results in the development of a fully functioning membrane attack complex (MAC), to create pores in plasma membranes. Ions and water influx cause cell osmotic swelling and finally its rupture. Notably, C9 has a molecular structure similar to perforin secreted by cytotoxic T lymphocytes (CTL) and NK cells. Additionally, C3 and C5 can be activated by unconventional proteases such as plasmin, cathepsin L, rennin, and thrombin (non-canonical pathway). The complement cascade and the stability of activated proteins are closely regulated to prevent effects on normal cells and to shorten its duration, since degradation products can affect healthy adjacent cells, for instance. Different circulating and membrane-bound regulators have been described so far [40]. C1 inhibitor (C1 INH) is a serin protease inhibitor of C1r and C1s. Normal mammalian membranes express several proteins that can block the assembly of C3 and C5 convertases. Membrane cofactor protein (MCP or CD46), type I complement receptor (CR1), and decay-accelerating factor (DAF) competitively bind to C3b or C4b on cell surfaces. Circulating factor H (FH) inhibits fragment Bb interaction to C3b (alternative pathway), as does circulating C4-binding protein (C4BP) for C4b. Factor I, a plasma serine protease, degrades C3b through proteolysis and needs MCP, FH, C4BP, and CR1 as cofactors. Resulting iC3b, C3d, and C3dg are recognized by phagocytes and B cells, thus not participating in complement activation. Normal host cells express CD59, which limits the assembly of MAC, as S protein (and other soluble proteins) does for circulating C5b,6,7 complexes. Although the complement system was at first considered crucial just for innate immunity, immune cells were demonstrated to express a complement receptor repertoire. The engagement of these receptors promotes an inflammatory reaction to remove damage. Hence, migration and activation of neutrophils and monocytes and pathogen or antigen phagocytosis can be coordinated by complement fragments receptors. Bone-marrow-derived cells (i.e., neutrophils, monocytes, eosinophils, erythrocytes, lymphocytes) typically exploit CR1 to internalize particles opsonized with C3b or C4b. The CR1 signaling pathway also enhances microbial clearance in phagocytes when Fcγ receptor is simultaneously engaged. C3a, C4a, and C5a are referred to as the anaphylatoxins since they can induce mast cell degranulation and histamine release. However, a wide range of cells express their G-protein coupled receptors: C3a receptor (C3aR) and C5a receptors 1 and 2 (C5aR1 and C5aR2). Additionally, complement can provide co-stimulatory signals to antigen-presenting cells (APCs), including B and T cells. Type 2 complement receptor (CR2) on B lymphocytes recognizes iC3b/C3dg/C3d fragments, facilitating the initiation of humoral immune responses by providing co-stimulatory signals and lowering the threshold of B cell receptor (BCR) signaling [41]. Anaphylatoxins support the classical inflammatory reaction by increasing vascular permeability to support cell adherence and tissue influx, smooth muscle contraction, immune cell chemotaxis, activation, and degranulation, and the release of proinflammatory mediators at the site of complement activation. In neutrophils, C5a encourages motility, adhesion to endothelial cells, and even the respiratory burst with subsequent reactive oxygen species (ROS) production at high concentrations [42]. It has become evident that C3a and C5aR1 recruitment on human APCs and T cells is mainly independent of serum-derived C3 and C5 during APC-T cell interaction. APCs can secrete C3 and C5 to be converted into C3a and C5a via C3/C5 convertase and factor B and D. C3aR and C5aR1 engagement on APCs triggers their maturation, expression of MHC II and costimulatory molecules, and cytokine generation to support T cells activation [43,44,45]. Therefore, it is now commonly acknowledged that complement serves as a necessary functional bridge between innate and adaptive immunity. 

## 3. Complosome: Definition and Roles

Core complement proteins C3 and C5, their activation fragments, their receptors, and their regulators have been found to be active intracellularly as the complosome [46,47,48]. Complosome components not only interact with each other but also with intracellular danger sensor and effector systems (inflammasomes, autophagosomes, and ribosomal machinery), controlling cell metabolism and general cell physiology (autophagy and gene expression) [49,50,51,52]. These components have been found in the cytoplasm, nucleus, lysosome, endoplasmic reticulum, and outer membrane of mitochondria. C3 and C5 can be activated by specific proteases or by C3/C5 convertases assembled beneath the plasma membrane or on the surface of subcellular compartments. Even if they are encoded by the same genes, extracellular proteins cannot compensate for cell-intrinsic elements. Complosome proteins might undergo post-translational modifications or have distinct structures (according to environmental conditions such as pH). Indeed, complosome components can also function in cellular compartments that are exclusively accessible to the cell-autonomous protein sorting machinery [53]. Interestingly, C3 component can be recruited intracellularly from the extracellular space or from the cell surface. In B cells, extracellular C3 translocation to the nucleus supports gene expression, whereas in CD4 T cells support IL-6 production [54,55]. Although it was first described in CD4 T cells, the complosome has been discovered in a wide range of immune and non-immune cells [56]. Cathepsin L (CTSL) steadily cleaves at low-level lysosomal C3 in circulating CD4 T cells. Then, lysosomal C3aR sustains mammalian target of rapamycin (mTOR) activation (survival signal). By keeping Jagged-1 away from Notch-1, CD46 prevents Notch-1-driven activation in resting CD4 T cells. The rapid translocation of C3b to the cell surface, where it binds CD46, is triggered by stimulation of the T cell receptor (TCR). Not only does autocrine CD46 stimulation release Jagged-1 to facilitate Notch-1 activation, but it also triggers several crucial steps necessary for T cells to release IFN-γ and develop into T cell helper type 1 (Th1) [57,58]. More, CD46 promotes gene expression of glucose (GLUT-1) and neutral ammino acid (LAT-1) transporters and triggers glycolysis, oxidative phosphorylation (OXPHOS), and fatty acid metabolism. Additionally, CD46 seems to regulate cholesterol synthesis by starting mTOR complex 1 (mTORC1) assembly at the lysosomal surface [59]. Two CD46 isoforms exist with different cytoplasmic signaling domains: CYT-1 and CYT-2. After its stimulation, γ-secretase complex releases CYT-1 and CYT-2, which translocate to the nucleus. However, they do not work as transcription factors because they lack a DNA binding site. Finally, CYT-1 and CYT-2 modulates gene expression (nutrient channels or metabolic enzymes). DNA–histone interactions apparently can depend on C3. CD46-induced intracellular C5 activation enhances oxygen mitochondrial metabolism (through mitochondrial C5aR1) and the assembly of a canonical NLR family pyrin domain containing 3 protein (NLRP3) inflammosome (IL-1β secretion). The contraction phase of Th1 response is also influenced by the complosome since they express C5aR2 intracellularly and on their surface. Its engagement by C5a or its desarginated version (C5a-desArg) negatively regulates inflammasome activity, reduces the release of IFN-γ in favor of IL-10, and suppresses Th1 activity [60]. During CD8 T cells’ activation, CD46 augments the synthesis of IFN-γ and granzyme B, and fatty acid metabolism through intracellularly generated C3b [61]. In human B cells, internalized C3 and C3a might control DNA packaging since they can enter the nucleus and bind histones. Complosome contributions to cytokine and antibodies generation remain unmapped, so far. Human monocytes and neutrophils are characterized by a continuous transcription of C3 and C5. The mitochondrial C5a/C5aR1 axis has been shown to reverse electron chain flux toward ROS production and aerobic glycolysis, reducing ATP/cAMP production and OXPHOS [49]. Surprisingly, apoptotic cells are bound by C1q before their phagocytosis. This seems to limit NLRP3 activity and macrophage polarization toward the M1-phenotype to grant a non-inflammatory clearance of apoptotic cells [62]. Unlike M2 macrophages, mitochondrial C5aR1 inhibition leads to reduced IL-10 production. Therefore, mitochondrial function and fitness have been suggested to be maintained by the complosome [63]. Autophagic machinery can be under the control of the complosome as well. Independently of the activation of its fragments receptors, C3 can directly interact with autophagy-related 16-like 1 protein (ATG16L1), thus controlling autophagy [64]. Complosome components have been described in different types of non-immune cells even if their contribution remains not utterly clear [65]. It can orchestrate responses against infectious and non-infectious stressors. In the presence of oxidative stress, C3-regulatory factor H-related protein 3 (FHR-3) increases intracellular complement activation and the release of pro-inflammatory cytokines, while mitochondrial C3aR decreases mitochondrial respiration in human retinal epithelial cells [66,67]. Hypoxia-inducible factor 1α (HIF1α) can be under the control of the CYT-2 domain of CD46 in bladder epithelial cells, where hyperactivation of MYC-mediated proliferation might be associated with malignant transformation [68]. Beyond the role of the extracellular complement system in human diseases, growing evidence suggests that the complosome can also be involved in several pathological conditions [69]. Increasing data suggest that variations in complosome activities in endothelial and tubular kidney cells can affect organ physiology or contribute to damage. A single study investigated its possible function in kidney glomerular endothelial cells. FH absence led to spontaneous cytoskeletal remodeling, cellular layer dysfunction, loss of barrier function, and heightened cell proliferation resulting in enhanced angiogenic potential [70]. Gene analysis revealed that FH deficiency alters the expression of several genes. Nuclear translocation of transcription factor nuclear factor kappa-light-chain enhancer of activated B (NF-kB) is triggered by the lack of FH, thus perturbing cellular function. In contrast to glomerular endothelial cells, kidney tubular cells do not show detectable levels of intrinsic FH in the steady state. According to this finding, FH knockdown did not alter the transcriptional program or cell behavior of tubular cells. Intracellular C5 and C5aR1 in kidney-infiltrating macrophages seem to play a crucial role in folic-acid-induced tubular cell injury. Reduced renal fibrosis was noted in mice with macrophages deficient for C5 and C5aR1. Single-cell RNA sequencing revealed that renal C3 and C5 expression was raised after mice unilateral ureteral obstruction, especially in tubular cells [71].

## 4. Complement and Kidney Diseases

The deposition of complement fragments or locally produced ones are detrimental for kidneys. Alternative pathway dysregulation is responsible for a plethora of kidney diseases. Genetic or acquired disorders result in its erroneous or prolonged activation [72]. Hemolytic uremic syndrome (HUS) is defined by platelet thrombi in the vessels of the kidneys and other organs provoking anemia, thrombocytopenia, and renal injuries (up to end-stage renal disease). Unlike HUS associated with Escherichia coli toxins (STEC-HUS), atypical HUS (aHUS) can occur through abnormalities in the complement cascade that are either genetic or acquired, as autoantibodies (anti-factor H). Affected individuals are extremely vulnerable to complement activation on endothelial cells after environmental hints (such as infections, drugs, pregnancy, etc.), leading to microangiopathic and renal injury [73,74,75]. After kidney transplantation, primary aHUS is linked to a high likelihood of recurrence and poor outcomes [76]. Genetic anomalies in the system proteins or its regulators are found in 40–60% of cases, with less than 20% being familial [77]. Genetic mutations are responsible for deficiency or dysfunction in FH (most frequently), FI, or CD46 in aHUS [78,79,80]. Rarely, gain-of-function variants in FB and C3 are identified, which prevents their degradation. Thrombomodulin (THBD) binds to C3b and FH on endothelial cells and supports C3b inactivation by FI and C3a/C5a inactivation through procarboxypeptidase B. Variants in THBD and factor-H-related (FHR) proteins can also contribute to aHUS [81,82]. Alterative pathway dysregulation has been found approximately in 50–70% of patients with C3-glomerulopathy (glomerular C3 deposition) and immune complex-associated membranoproliferative glomerulonephritis (glomerular Ig and complement deposition). Genetic variants have been described for C3, FB, FH, FI, CD46, and TBHD [83]. Alternative pathway C3 and C5 convertases are targets of autoantibodies (nephritic factors C3NeF and C5NeF), which prolong their half-lives. They are the most common autoantibodies in these diseases [84]. Of note, disease recurrence after kidney transplantation highly affects these patients. Histological findings of membranous nephropathy (MN) include glomerular basal membrane thickening and the deposition of IgG, antigens, and complement components. C3 and C5b-9 are consistently present alongside IgG in the subepithelial space [85]. Each of the complement pathways can be involved in the MN; however, none of them appears to be exclusive [86]. Secondary MN can develop in association with viral infections, drug toxicity, or systemic lupus erythematosus (SLE). Autoantibodies against phospholipase A2 receptor (PLA2R) and thrombospondin type-1-domain-containing protein 7A (THSD7A) define primary MN. Recently, many other proteins have been identified as possible autoantigens, such as contactin 1, semaphoring 3B, transforming growth factor-β receptor 3, and netrin G1 [87]. Positive staining for C5b-9, C3d, FB, and properdin has been observed in kidney biopsies from patients with anti-neutrophil cytoplasmic autoantibody (ANCA)-associated vasculitides (AAV), even though immune complex deposits are typically absent [88]. Acute postinfectious glomerulonephritis (APIGN) can occur after an extrarenal infection, such as skin or pharynx infection by group A β-hemolytic Streptococci. Despite glomerular deposition of immune complexes, there is a selective activation of the alternative pathway. Most of the patients have normal serum C4 levels and reduced C3 levels [89]. Predominant activation of the alternative and lectine pathways is noted in the pathogenesis of IgA nephropathy [90]. Indeed, C3, properdin, C4d, MBL, and C5b-9 are deposited in the glomeruli, unlike C1q [91]. Inflammation in the kidney is initiated by immune complexes containing galactose-deficient IgA1. A relationship between the alternative pathway and disease severity has been unveiled: the levels of Ba fragments (smaller activation fragment of FB) directly correlate with the degree of proteinuria and indirectly with estimated glomerular filtration rate (eGFR) [92]. One of the toughest manifestations of SLE is lupus nephritis (LN). Upon immune complex glomerular accumulation, C1q binding allows the classical pathway to begin. Evidence posits the involvement of the alternative pathway in LN pathogenesis [93]. In contrast to individuals with LN that was in remission, patients with active LN had higher plasma concentrations of Bb, C3a, C5a, and sC5b-9, as well as lower plasma levels of C1q and C3 [94]. Furthermore, one-year immunosuppressive response rates for LN patients with biopsies demonstrating glomerular deposition of C3 without C1q and C4 were lower, and they were more likely to have renal disease progression. Compared to SLE patients without clinical evidence of renal involvement or healthy controls, serum levels of FH were considerably lower in LN patients. These levels were also inversely correlated with the SLE disease activity index and renal activity index scores [95]. Additional kidney diseases that show some evidence of complement activation include anti-GBM disease (Goodpasture’s), diabetic nephropathy, and cryoglobulinemia [96,97,98,99].

## 5. Complement and Kidney Transplantation

Kidney transplantation offers a better survival rate and quality of life than dialysis to patients with end-stage renal disease (ESRD). Kidney ischemia-reperfusion injury (IRI), delayed graft function (DGF), T- and B-cell mediated rejection, and chronic allograft injury can affect long-term graft survival and could lead to graft loss [100,101,102]. IRI is an unavoidable process after transplantation, which can trigger the immunogenicity of the graft and can raise the risk of acute rejection [103]. Different events occur during IRI: transcriptional reprogramming, activation of apoptosis, necrosis and necroptosis, endothelial dysfunction, and activation of innate and adaptive immune responses [104,105]. The rationale for the creation of an immune microenvironment is for the clearance of injured cells and for subsequent tissue repair [106,107,108]. Locally generated component proteins seem to be more predominant during IRI than circulating ones. C3 silencing with small interfering RNA (siRNA) could ameliorate IRI [109]. The lectin pathway has recently been identified as one of those most heavily involved, despite earlier studies pointing at the alternative pathway. Upon IRI, tubular cells increase the production of collectin-1 (CL11), a soluble lectin molecule. At the ischemic sites, CL11 binds to stressed cells, thus initiating complement cascade via MASPs [110,111]. While overexpression of human CD55 and CD59 yielded better renal function, their deficiency left mice more sensitive to IRI [112,113,114]. Besides MAC insertion, anaphylatoxins are generated, whose receptors are expressed on different cells. Their priming, costimulatory receptors expression, and antigen presentation are enhanced by C3a and C5a on antigen-presenting cells (APCs). Complement fragments support T cells’ proliferation and differentiation and B cells’ response. IgM to IgG switch is encouraged by complement receptor 2 (CR2) on B cells upon their activation [115]. C5aR1 has been found to play a role in fibroblast proliferation and activation after IRI [116]. Pericytes and endothelial cells also play a significant role in renal fibrosis and scar formation after kidney damage, also because of endothelial-to-mesenchymal transition (EndMT). EndMT was limited by blocking the complement system, and scar formation and collagen deposition were reduced with C1q treatment [117,118,119]. C5aR engagement on tubular cells promotes aberrant DNA methylation, altering the expression of genes involved in the cell cycle, DNA damage control, and signalling. BCL9, CYP1B1, and CDK6 were silenced, while p53 and p21 (cell cycle arrest) were upregulated [120,121]. The gene Klotho is known to exert anti-senescence and anti-fibrotic effects, and its expression was reduced in a swine model of IRI through complement activation [122]. The occurrence of delayed graft function (DGF) is directly influenced by IRI. Graft recipients with DGF present significant complement activation. Indeed, serum C5b-9 levels were higher compared to recipients with early graft function, making them ideal biomarkers for predicting graft function and long-term outcomes. Graft biopsies further uncovered MAC and C3d deposition [123,124]. During reperfusion, C5b-9 levels were noticeably higher in deceased-donor kidney transplantation than living ones [125]. Therefore, it has been suggested that the complement cascade initiates in deceased donors prior to organ procurement because of impaired homeostasis, unstable hemodynamic, and circulating DAMPs from injured cells. Graft survival is threated by T-cell mediated (TCMR) and antibody-mediated rejection (AMR). The complement system influences T cell priming and activation both directly and indirectly through APCs. In APCs, C5aR1 signaling drives the expression of MHC and co-stimulatory molecules and the production of cytokines and growth factors [126,127]. The role of the complosome in T and B cells has already been discussed earlier. After kidney transplantation, recipients can develop donor-specific antibodies (DSA), including anti-ABO and anti-HLA as part of their humoral immunity. These immunoglobulins bind to endothelial cells during AMR, initiating the complement classical pathway [128]. In turn, anaphylatoxins potentiate inflammation, whereas MAC hurts the integrity of blood vessels because of CDC [129]. In the past years, C4d deposition had been a histological criteria for AMR [130]. Finally, interstitial fibrosis/tubular atrophy (chronic allograft nephropathy) can be due to IRI, drug toxicity, rejection, or infections as a result of maladaptive repair after graft damage. Fibroblast activation leads to scar formation, thus affecting graft activity.

## 6. Overview of Complement System in Cancer

The complement system represents a robust component of the immune system against microbes and “non-self” cells. Besides cancer cells, the tumor microenvironment (TME) consists of different immune (i.e., B and T cells, tumor-associated macrophages, neutrophils, myeloid-derived suppressor cells) and non-immune cells (i.e., cancer-associated fibroblasts—CAFs), and extracellular elements. Tumor cells gradually induce phenotypic changes in the TME by releasing soluble factors (cytokines, growth factors, enzymes) and recruiting cells to foster its growth, progression, and metastasis [131]. Complement system elements have been proved to be present in the TME of various human malignancies. Interestingly, most of data from in vitro studies or animal models have yielded sometimes contradictory results; therefore, it is now acknowledged that the effects of complement are context-dependent across cancer types [132,133,134]. Considering the extensive tumor vasculature, most of the complement proteins produced in the liver are easily accessible in the TME, where they can also be secreted locally by cancer, stromal, and immune cells [135]. For instance, CAFs are a major source for complement proteins in melanoma (C1s, C1r, C3, C4a, FB, and C1inh) and breast cancer (C3 and C3a) [136,137]. Complement fragments can be detected along tumor vessels, although the main pathway of its activation in the TME is still unclear. Cascade-independent proteases can activate C3 and C5, such as urokinase-plasminogen activator (uPA) in mouse squamous carcinogenesis [138]. Conversely, tumor cells adopt different mechanisms to escape complement-dependent cytotoxicity (CDC) triggered by MAC. The expression of either membrane-bound (CD21, CD35, CD46, CD55, CD59) or soluble (FH, factor H-like protein) regulators can be increased [139]. The assembly and stability of MAC can be apparently counteracted by heat shock protein 90 (HSP90) together with mortalin [140]. In addition, evidence suggests that the complement system can support tumor progression by orchestrating the composition of the TME as well as controlling cancer cell proliferation, epithelial-to-mesenchymal transition (EMT), angiogenesis, metastasis, and stemness [141]. The accumulation and immunosuppressive effects of tumor-associated macrophages (M2-TAMs) can be controlled by C3a-C3aR signaling [142]. The recruitment and differentiation of MDSCs in the TME is triggered by C5aR-mediated pathways. MDSCs are able to secrete a huge variety of immunomodulators, such as arginase-1 (Arg-1), IL-10, transforming growth factor-β (TGF-β), and express immune checkpoint molecules, such as cytotoxic T lymphocyte antigen 4 (CTLA4) and programmed death-ligand 1 (PD-L1). C5a shifts CD4 T cells toward T helper 2 (Th2) or regulatory T cells (T regs) to further limit T cells’ activities. Blocking C5aR signaling reduced T regs generation and TGF-β production [143,144]. Neutrophils accumulation and their polarization toward a pro-tumorigenic phenotype (N2-TANs) are associated with complement activation in the TME. In ovarian cancer, neutrophils can affect antitumor T cells’ response upon C3 activation [145]. C3aR could regulate the recruitment of N2-TANs through the neutrophil extracellular traps (NETs) [146]. In a mouse breast cancer model, CAFs are thought to be controlled by C3a-C3aR signaling: their activation leads to the production of pro-tumorigenic cytokines (TGF- β), EMT, then metastasis [137]. Besides modulating the composition of the TME, the complement system can directly enhance cancer growth. In particular, ovarian cancer cells’ proliferation increased in vivo in the presence of C3aR and C5aR agonists, whilst it decreased with their antagonists [147]. Ovarian cancer cells secrete C3a, which can downregulate E-cadherin via transcription factor TWIST, thus promoting EMT [148]. In hepatocellular carcinoma, the C5a-C5aR axis reduces E-cadherin and claudin-1 expression by upregulating Snail and activating ERK1/2 pathways [149]. Its blockade alters the migration of lung cancer cells [150]. The proangiogenic role of complement has been described in a mouse model of ovarian cancer. C3 inhibition or C5aR knockout significantly hamper the expression of VEGF and the activity of endothelial cells [151]. By controlling stromal cells in the TME, the complement system can indirectly support angiogenesis. Supporting angiogenesis, EMT, extracellular matrix degradation (ECM), and immunosuppression, the complement system can therefore contribute to metastases. Cancer stem cells (CSCs) are characterized by self-renewal and tumorigenicity and are thought to be responsible for cancer recurrence [152]. SOX2 was reported to upregulate CD59 expression on CSCs, thus preventing CDC [153]. Roumenina et al. analyzed the gene expression of the main complement elements in 30 tumor types. They recognized a significant expression of C3 and classical pathway components (C1QA, C1QB, C1QC, C1R, C1S, C4A, and C2) in all cancer types. Alternative pathway complement factors B and D (FB and FD) are upregulated with low expression in chromofobe renal cell carcinoma (chRCC), though. Low expression of terminal elements (C8A, C8B, and C9) was detected in all cancer types, whereas complement regulators (C1inh, FH, FI CD46, CD55, and CD59) are highly expressed in most cancers. Using the TCGA database, they next evaluated the effect of gene expression of classical and alternative pathways on patient overall survival. In the first cancer group (including prostate adenocarcinoma-PRAD), strong gene expression was associated with good prognosis. C3 expression was linked to longer overall survival in the second group (i.e., chromophobe RCC- KICH). Poor prognosis characterized the third cancer group (i.e., clear cell RCC—KIRC). The fourth group includes malignancies (papillary RCC—KIRP) for which a gene expression study did not show any clear clinical impact [132]. Considering the crucial role of the complement system in tumor biology, different therapeutic strategies have been adopted so far. Monoclonal antibodies have been introduced in cancer therapy, exploiting CDC as a mechanism of action. These IgG1 antibodies can activate the complement cascade, thus triggering tumor cell killing after binding to their cancer specific epitope as CD38 (daratumumab) or HER2 (pertuzumab) [154,155]. Cancer cells soon adopt various mechanisms to drive resistance, such as the expression of membrane-bound complement regulatory proteins (mCRPs). Therefore, bi-specific antibodies targeting both a tumor antigen and an mCRP were developed to prevent the negative effects of non-specific targeting of a complement regulator. In a mouse model of Burkitt’s lymphoma, increased survival was reached with antibodies targeting CD20 and CD55 or CD59 [156]. There are limited data about the role of the complosome in cancer cells. In a mouse model of colon carcinoma, C3 is cleaved intracellularly by cathepsin L and B. Tumor-derived C3a triggers the C3a-C3aR-PI3K pathways on TAMs to limit T cell activity. In this model, the efficacy of anti-PD-L1 therapy was enhanced after C3 deletion [142]. As in T cells, the complosome is theorized to regulate metabolic processes. Therefore, future investigation can provide better insight in this field.

## 7. Complement System in Kidney Cancer

**Figure 2:**
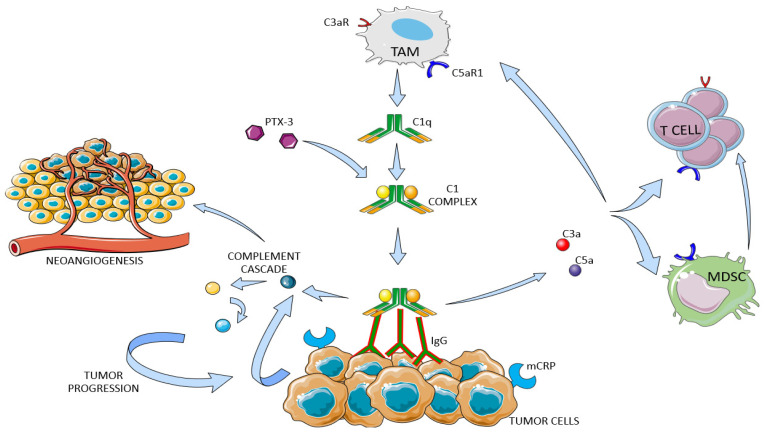
Putative complement network in ccRCC TME. Tumor-associated macrophages (TAM) secrete C1q, which then assemble with C1r and C1s to form functionally active C1 complex. The cascade initiation depends on IgG deposition on tumor cells or PTX-3. Anaphylotoxins C3a and C5a support MDSCs’ recruitment and T cells’ exhaustion. Besides promoting modulating neoangiogenesis and immunoglogosis, the complement system can directly promote tumor growth.

The role of the complement system in renal cell carcinoma (RCC) has been recently explored (Figure 2).

The bioinformatics dataset revealed that clear cell (KIRC) and papillary (KIRP) renal cell carcinoma upregulate most of complement genes relative to normal kidney tissue. In the TME of ccRCC, TAMs produce C1q, whereas cancer cells synthetize C1r, C1s, C4, and C3. C1 complex assembly enables the complement cascade to start [157,158]. Factor H (FH) limits the cascade at the level of C3 convertase. In accordance with these findings, higher FH mRNA levels correlate with increased survival in RCC patients [159]. Considering the high mRNA and protein levels of C3 and fibronectin-1, Dong and colleagues further supported their role in RCC tumorigenesis and progression [160]. In this scenario, C3aR and C5aR1 signaling were assessed in a murine model of RCC. Tumor growth was slowed down by genetic C3aR deficiency or C5aR1 pharmacological blockade. C3aR knockout increased INF-γ production by CD8 T cells to the greatest extent and decreased the expression of inhibitory receptors (Pdcd1, Ctla4, and Btla) [161]. The non-canonical and cascade-independent role of C1s was investigated in ccRCC. Transcriptional programs were affected upon its inactivation, as well as reduced cell proliferation and viability. It has been hypothesized that C1s can interact with HMGB-1, which binds nucleosomes, histones, and transcription factors in the nucleus. In complement-rich tumors, C1s can also exert its enzymatic activity intracellularly toward its canonical effectors. Elevated serum levels of C4d were associated with poor prognosis in ccRCC patients. In any of the individuals studied, there was no evidence of hypocomplementemia, indicating no C4 consumption in the blood [162]. RNA-sequencing analysis has recently stated that a high alternative complement pathway signature (ACPS) correlates with higher staging and grading, and worse survival in RCC. Moreover, high-ACPS tumors highlight significant accumulation of TAMs and suppressive cytokines (CCL5, IL10, CXCL8, CCL17, and CCL22). Notably, this retrospective study has suggested that ACPS can predict the efficacy of tyrosine kinase inhibitor and immune checkpoint inhibitor (TKI+ICI) combination therapy (survival benefit in low-ACPS) [163].

As mentioned above, pentraxin-3 (PTX-3) can activate the classical and lectin pathways (binding with C1q and MBL) or regulate the alterative pathway (binding with FH) [164,165,166]. Renal cancer cells and ccRCC-derived tissues showed an increased expression of PTX-3 in association with C1q, C3a, C5a, C3aR, C5aR1, and CD59 overexpression. Through the activation of the classical pathway of the complement cascade and the subsequent production of pro-angiogenic factors (C3a and C5a), PTX-3 was able to regulate immunoflogosis in the ccRCC TME. The authors retrospectively analyzed the PTX-3 serum levels of patients undergoing nephrectomy as well. At first, they realized that pre-operative levels of ccRCC patients were higher than non-neoplastic patients. Higher preoperative levels were also dramatically associated with higher Furhman grading, lymph node and distant metastases at the time of diagnosis, and lower patients’ long-term survival. Curiously, PTX-3 levels considerably decreased after nephrectomy, strengthening the relationship between its intra-tumor production and serum levels [167]. Our research group has recently evaluated the association between mucin 1 (MUC1) expression, complement system activation, and immune cell infiltration in ccRCC specimens. Most human malignancies exhibit MUC1 overexpression or its abnormal glycosylation. This membrane glycoprotein is involved in glucose and lipid metabolic reprogramming in ccRCC, and reduced patients’ cancer-specific (CSS) and progression-free survival (PFS) were noted in cases of higher levels of CA15-3 (serum form of MUC1) [168,169]. ccRCC samples with increased expression of MUC1 (MUC1^H^) show extensive C1q deposition, which co-localizes with PTX-3. In addition, anaphylatoxin receptors and CD59 were markedly high in MUC1^H^ samples. As concerns the composition of the TME in MUC^H^ specimens, increased number of mast cells and M2-TAMs (CD68+ CD163+ IDO1+) and reduced CD8 T cells were revealed. Of note, MUC1 and PD-L1 expression were negatively correlated [170,171].

## 8. Clinical Applications

Eculizumab (humanized anti-C5 monoclonal antibody) was approved in 2011 for the treatment of aHUS, and after its introduction in clinical practice, the outcome of this disease is significantly improved. Upon binding to C5 with high affinity, eculizumab prevents the production of C5a and MAC deposition while maintaining early complement functions (opsonization and immune clearance) [172]. Different clinical trials have investigated the effects of complement inhibition (C1, C3, and C5) to prevent DGF and acute rejection (especially AMR) after kidney transplantation. Two small, randomized clinical trials (NCT01403389 and NCT01919346) and the results of the PROTECT study (NCT0214518) indicate that although peritransplant administration of Eculizumab is safe in transplant recipients of deceased donor kidney, it has no efficacy in preventing the development of DGF [173,174]. Surprisingly, pediatric patients experienced earlier graft function and developed less chronic glomerulopathy and less arterial hyalinosis after eculizumab treatment [175]. In the NCT02134314 trial, C1 inhibitor (C1-esterase inhibitor) did not provide significant benefits over a placebo in the occurrence of DGF, but the time of dialysis was considerably reduced and long-term eGFR was better in treated individuals [176,177]. When compared to the control group (plasma exchange protocol), eculizumab reduced the incidence of AMR in sensitized recipients of living donors’ grafts (NCT00670774) [178]. Eculizumab alone did not improve eGFR within 3 months in patients who already developed AMR (NCT01895127). Compared to control, none of the highly HLA-sensitized study participants developed DGF or AMR when they received C1 inhibitor [179]. Future research is needed to demonstrate that complement therapy significantly improves graft survival when compared to standard treatments.

Currently, the investigation of the complement components’ roles in different tumors is still in its early stages. Emerging evidence shows how this system is important in tumor-associated immune responses, tumor cell biology, and stemness pathways.

To date, there are no ongoing clinical trials examining complement-targeted therapy for cancer, including RCC, although animal studies have shown the effectiveness of anticomplement molecules in reducing tumor size. An interesting application of complement-targeted therapy in cancer treatment is its association with immune checkpoint inhibitors (ICIs). While ICIs increase the T-cell-mediated antitumor immune, anticomplement molecules decrease the infiltration of myeloid-derived suppressor cells (MDSCs) that assist cancer cells in escaping immune surveillance. The synergistic effect of this combination therapy would therefore result in a reduced MDSC-induced T cell suppression, and enhanced cytotoxic T cell function.

## 9. Conclusions

Accumulating evidence about the role of complement in human diseases paved the way to the development of complement-directed therapies [180,181]. Complement inhibition has also opened intriguing approaches for the treatment of several different renal diseases and allograft recipients in which complement activation is implicated to varying degrees. In-depth understanding of the context-dependent role of complement in cancers might provide further strategies for immunotherapy. When developing drugs, it is of utter importance to consider which component to block effectively without hampering patients’ defenses against infections. Future perspectives in this field might include the role of the complosome and locally produced elements in kidney disorders.

## Data Availability

No new data were created.
